# Screen for Chemical Modulators of Autophagy Reveals Novel Therapeutic Inhibitors of mTORC1 Signaling

**DOI:** 10.1371/journal.pone.0007124

**Published:** 2009-09-22

**Authors:** Aruna D. Balgi, Bruno D. Fonseca, Elizabeth Donohue, Trevor C. F. Tsang, Patrick Lajoie, Christopher G. Proud, Ivan R. Nabi, Michel Roberge

**Affiliations:** 1 Department of Biochemistry and Molecular Biology, University of British Columbia, Vancouver, British Columbia, Canada; 2 Department of Cellular and Physiological Sciences, University of British Columbia, Vancouver, British Columbia, Canada; 3 School of Biological Sciences, University of Southampton, Southampton, United Kingdom; Karolinska Institutet, Sweden

## Abstract

**Background:**

Mammalian target of rapamycin complex 1 (mTORC1) is a protein kinase that relays nutrient availability signals to control numerous cellular functions including autophagy, a process of cellular self-eating activated by nutrient depletion. Addressing the therapeutic potential of modulating mTORC1 signaling and autophagy in human disease requires active chemicals with pharmacologically desirable properties.

**Methodology/Principal Findings:**

Using an automated cell-based assay, we screened a collection of >3,500 chemicals and identified three approved drugs (perhexiline, niclosamide, amiodarone) and one pharmacological reagent (rottlerin) capable of rapidly increasing autophagosome content. Biochemical assays showed that the four compounds stimulate autophagy and inhibit mTORC1 signaling in cells maintained in nutrient-rich conditions. The compounds did not inhibit mTORC2, which also contains mTOR as a catalytic subunit, suggesting that they do not inhibit mTOR catalytic activity but rather inhibit signaling to mTORC1. mTORC1 inhibition and autophagosome accumulation induced by perhexiline, niclosamide or rottlerin were rapidly reversed upon drug withdrawal whereas amiodarone inhibited mTORC1 essentially irreversibly. TSC2, a negative regulator of mTORC1, was required for inhibition of mTORC1 signaling by rottlerin but not for mTORC1 inhibition by perhexiline, niclosamide and amiodarone. Transient exposure of immortalized mouse embryo fibroblasts to these drugs was not toxic in nutrient-rich conditions but led to rapid cell death by apoptosis in starvation conditions, by a mechanism determined in large part by the tuberous sclerosis complex protein TSC2, an upstream regulator of mTORC1. By contrast, transient exposure to the mTORC1 inhibitor rapamycin caused essentially irreversible mTORC1 inhibition, sustained inhibition of cell growth and no selective cell killing in starvation.

**Conclusion/Significance:**

The observation that drugs already approved for human use can reversibly inhibit mTORC1 and stimulate autophagy should greatly facilitate the preclinical and clinical testing of mTORC1 inhibition for indications such as tuberous sclerosis, diabetes, cardiovascular disease and cancer.

## Introduction

The cellular processes linked to growth are tightly modulated by nutrient levels. Anabolic functions such as ribosome biogenesis and protein synthesis are inhibited under conditions of nutrient limitation, while catabolic pathways such as autophagy are activated. Autophagy, a process of cellular self-eating, can temporarily compensate for lack of extracellular nutrients by engulfing cytoplasmic components within double-membraned autophagosomes, degrading them by fusion with lysosomes and releasing building blocks for macromolecular synthesis [1], [2]. Mammalian target of rapamycin complex 1 (mTORC1) plays a critical role in coupling nutrient sensing to these anabolic and catabolic processes [3]. When nutrients are available, mTORC1 is switched on and negatively regulates autophagy while positively regulating ribosome biogenesis and protein synthesis [4], [5]. Conversely, nutrient limitation turns off mTORC1 signaling, leading to inhibition of cell growth and stimulation of autophagy.

mTORC1 is a protein complex composed of the serine/threonine kinase mTOR, the scaffolding protein raptor and mLST8 [3]. mTORC1 controls the initiation step of protein synthesis through the phosphorylation of eukaryotic initiation factor 4E-binding proteins (4E-BPs) [6], [7] and of ribosomal S6 kinases (S6Ks) [8]. 4E-BPs are a family of small proteins that associate with eIF4E, an mRNA cap-binding protein. eIF4E, together with eIF4G and eIF4A form the eIF4F complex that recruits the small (40S) ribosomal subunit to the 5′-end of mRNA. 4E-BPs and eIF4G bind to overlapping regions in eIF4E such that binding of 4E-BPs to eIF4E precludes the binding of eIF4G and blocks recruitment of the ribosome to the message [3]. The binding of 4E-BP1 to eIF4E is blocked through mTORC1-dependent phosphorylation of multiple residues on 4E-BP1. mTORC1 also phosphorylates the S6Ks that in turn phosphorylate multiple translation components including eIF4B and ribosomal protein S6. However, the role of phosphorylation of these proteins in stimulating protein synthesis remains to be elucidated [9].

Studies in metazoans and lower eukaryotes indicate that TORC1 plays an important role in the control of autophagy. Deletion in *Drosophila* of TOR or Rheb, an activator of TORC1, enhances autophagy even under the nutrient-rich conditions in which autophagy is typically downregulated [10]. Conversely, deletion of *Drosophila* TSC2, an inhibitor of Rheb/TORC1 signaling, blocks autophagy induced by nutrient withdrawal [10]. In budding yeast, TOR has been proposed to inhibit autophagy through phosphorylation of the Atg1/Atg13 complex [11], which regulates the recruitment of proteins to, and development of, nascent autophagosomes [12]. Phosphorylation of Atg13 by TOR precludes the binding of Atg13 to Atg1, resulting in a marked decrease in the kinase activity of Atg1 [11]. A putative human homologue of Atg13 has been identified [13] that forms a complex with ULK1 and FIP200 that may be directly regulated by mTORC1 [14]. In keeping with genetic data, rapamycin, a specific inhibitor of mTORC1, induces autophagy in mammalian cells as well as in *S. cerevisiae* and *D. melanogaster* [11], [15]–[17].

In addition to promoting cell survival in starvation conditions, autophagy plays a key role in cellular homeostasis by degrading long-lived proteins, damaged organelles and abnormal protein aggregates whose accumulation can lead to cell death, muscular- and neuro-degenerative diseases and cancer [5], [18]. Defects in autophagy may contribute to tumorigenesis, by allowing the accumulation of damaged mitochondria, which can lead to genetic instability [5]. Autophagy is also frequently observed in dying cells, prompting the suggestion that it can constitute a death mechanism [18]. Therefore, defects in autophagy could also contribute to cancer cell survival [5].

Addressing the therapeutic potential of modulating mTORC1 signaling and autophagy in human disease requires active chemicals with pharmacologically desirable properties [2], [19], [20]. We have developed an assay to detect chemicals that cause a rapid increase in cellular autophagosome content. A screen of >3,500 approved and off-patent medications, as well as compounds with known pharmacological activity led to the identification of three drugs approved for use in humans (amiodarone, niclosamide and perhexiline) and the pharmacological agent rottlerin. Consistent with their ability to modulate autophagy, we show that these chemicals also control mTORC1 signaling. Rottlerin inhibits mTORC1 signaling via TSC2 while the other drugs inhibit mTORC1 signaling in a TSC2-independent manner. Transient exposure to niclosamide, perhexiline or rottlerin causes reversible inhibition of mTORC1 signaling and is not toxic to cells in conditions of nutrient and growth factor sufficiency. However, these drugs selectively kill cells in starvation conditions. Drugs already approved for human use that can reversibly inhibit mTORC1 signaling and stimulate autophagy are valuable pharmacological tools to evaluate the therapeutic potential of manipulating mTORC1 and autophagy in disease.

## Results

### Development of an automated microscopy screen for chemical modulators of autophagy

**Figure 1:**
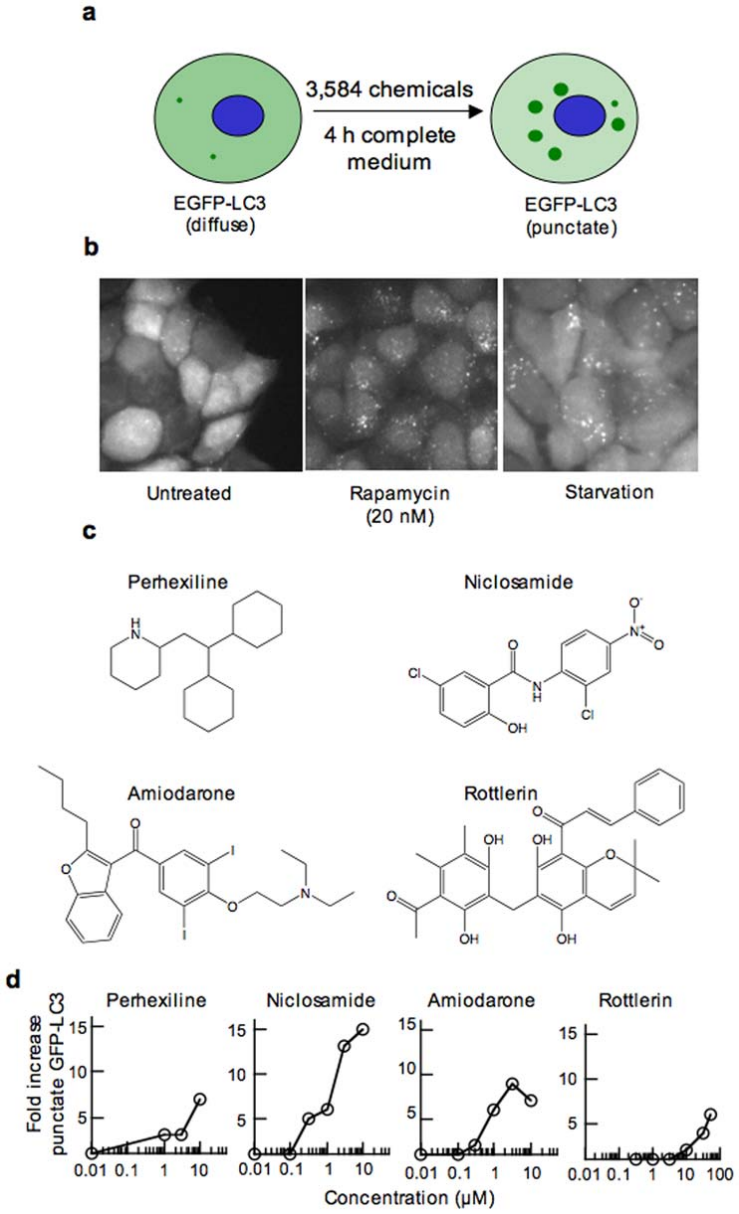
Cell-based assay for the identification of chemical modulators of autophagy and identification of amiodarone, niclosamide, perhexiline and rottlerin as modulators of autophagy. (a) Schematic representation of the EGFP-LC3 puncta formation assay used to screen for chemical modulators of autophagy. (b) Punctate EGFP-LC3 accumulation upon treatment with rapamycin or nutrient deprivation. MCF-7 cells stably expressing EGFP-LC3 were incubated without or with 20 nM rapamycin in complete cell culture medium or in medium lacking serum and amino acids for 4 h. EGFP-LC3 punctate staining was visualised by automated microscopy as described in Materials and Methods. (c) Chemical structures of perhexiline, niclosamide, amiodarone and rottlerin. (d) Quantitation of punctate EGFP-LC3 accumulation upon incubation with the active chemicals. MCF-7 cells stably expressing EGFP-LC3 were incubated for 4 h with different concentrations of the chemicals and EGFP-LC3 puntacte staining was quantitated using the automated microscopy assay.

Upon autophagy induction, the cytosolic Atg8 protein, also called LC3, is recruited to the membrane of nascent autophagosomes and controls autophagosome expansion [21]. LC3 is synthesised as a precursor protein whose C-terminus is cleaved by a cysteine protease to expose a glycine residue that is subsequently conjugated to phosphatidylethanolamine by a ubiquitin-like system [22]–[24]. To develop a screening assay for chemical modulators of autophagy, human breast cancer MCF-7 cells were stably transfected with a plasmid for expression of LC3 linked at its N-terminus to EGFP (Fig. 1a). In complete cell culture medium containing glucose, amino acids and serum, EGFP-LC3 fluorescence was largely diffuse throughout the cytoplasm with few dots denoting basal autophagosome formation (Fig. 1b). The number of EGFP-LC3 dots rapidly increased within 4 h exposure to the mTORC1 inhibitor rapamycin, or to amino acid- and serum-free medium (Fig. 1b; Supplementary Figure S1), conditions that are known to stimulate autophagy [25]. We wished to identify chemicals that, like rapamycin and starvation, also rapidly increase EGFP-LC3 punctate staining in cells maintained in nutrient-rich conditions, where autophagy is normally downregulated. The microscopy assay was automated using a high-content screening instrument programmed to detect and quantitate punctate EGFP-LC3 fluorescence. The Z-factor [26] for the assay was 0.55, appropriate for use in screening (Fig. S2). As demonstrated by the automated assay, withdrawal of amino acids and serum for 4 h caused a 3-fold increase in punctate EGFP-LC3 fluorescence intensity (Fig. S1b).

A collection of 3,584 drugs and pharmacologically active chemicals was tested at a concentration of ∼15 µM for 4 h in complete cell culture medium. Chemicals causing a ≥80% reduction in cell number were considered overtly toxic and were disregarded. Compounds that induced a ≥3-fold increase in punctate EGFP-LC3 intensity were designated as active (Fig. S2b). Four active chemicals, perhexiline, niclosamide, amiodarone and rottlerin (Fig. 1c), showed concentration-dependent activity ranging from 7- to 15-fold increased punctate EGFP-LC3 fluorescence intensity at their optimal concentration (Fig. 1d).

Amiodarone has previously been found to reduce the accumulation of expanded polyglutamine aggregates [27] and to enhance the clearance of mutant huntingtin and A53T α-synuclein in human cells [28], likely through the stimulation of autophagy. Rottlerin has recently been reported to induce autophagy in a PKC∂-independent manner in fibrosarcoma cells [29]. To our knowledge, neither niclosamide nor perhexiline have been previously reported to modulate autophagy.

### Characterization of amiodarone, rottlerin, niclosamide and perhexiline as stimulators of autophagy

**Figure 2:**
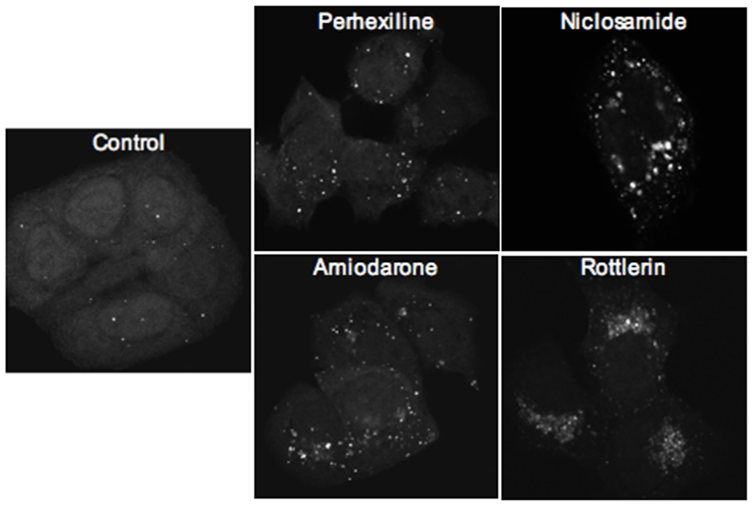
Visualization of punctate EGFP-LC3 in cells treated with autophagy-modulating compounds. MCF-7 cells stably expressing EGFP-LC3 were treated for 4 h with DMSO (control), 10 µM perhexiline, 10 µM niclosamide, 50 µM amiodarone or 3 µM rottlerin. Photographs show stacks of confocal sections spanning the entire cell thickness.

To verify that the punctate EGFP-LC3 fluorescence induced by the four chemicals represented autophagosome formation rather than, for instance, fluorescent drug precipitates, EGFP-LC3 fluorescence was examined at higher resolution by laser confocal microscopy. As expected, non-treated cells showed diffuse EGFP-LC3 fluorescence with few punctate structures (Fig. 2). Incubation with perhexiline, niclosamide, amiodarone or rottlerin for 4 h induced the appearance of a large number of EGFP-LC3-labeled cytoplasmic vesicles consistent with autophagosome formation (Fig. 2).

To ensure that punctate fluorescence detected in drug-treated cells was due to modulation of autophagy, we next monitored EGFP-LC3 processing and degradation. Recruitment of LC3 to nascent autophagosomes involves its proteolytic cleavage and lipidation [30]. This processing step, which also occurs with EGFP-LC3, yields a polypeptide (EGFP-LC3II) with increased electrophoretic mobility. When autophagosomes fuse with lysosomes, EGFP-LC3II is degraded by lysosomal hydrolases and the labile LC3II moiety is degraded faster than the more stable EGFP moiety, leading to transient accumulation of EGFP, which is also eventually degraded [31]. The EGFP-LC3II and EGFP bands can therefore be considered as characteristic proteolytic intermediates in autophagy. A time-dependent accumulation of free EGFP was readily observed upon incubation of MCF-7 cells stably expressing EGFP-LC3 with rapamycin (Fig. S1b), consistent with the observed time-dependent increase in punctate EGFP-LC3 (Fig. S1a). Similarly, serum deprivation caused an accumulation of free EGFP within 1 h (Fig. S3a). To verify that the four active chemicals indeed modulated autophagy, we examined the appearance of these bands following incubation with a range of concentrations of the chemicals and for different times.

**Figure 3:**
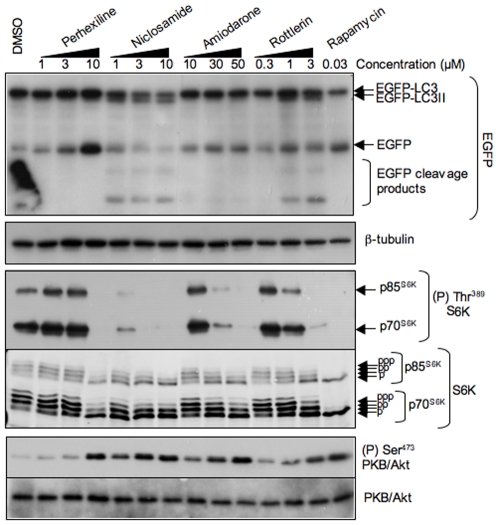
Concentration-dependent stimulation of EGFP-LC3 processing and inhibition of mTORC1 signaling by perhexiline, niclosamide, amiodarone and rottlerin. MCF-7 cells stably expressing EGFP-LC3 were treated for 4 h with chemicals at the indicated concentrations or with 20 nM rapamycin. EGFP-LC3 processing was monitored by probing lysates with anti-GFP antibody, mTORC1 activity by probing lysates with antisera against phosphorylated (Thr^389^) S6K and total S6K, and mTORC2 activity with antisera against phosphorylated (Ser^473^) PKB/Akt and total PKB/Akt. β-tubulin immunostaining was used as a protein loading control.

**Figure 4 pone-0007124-g004:**
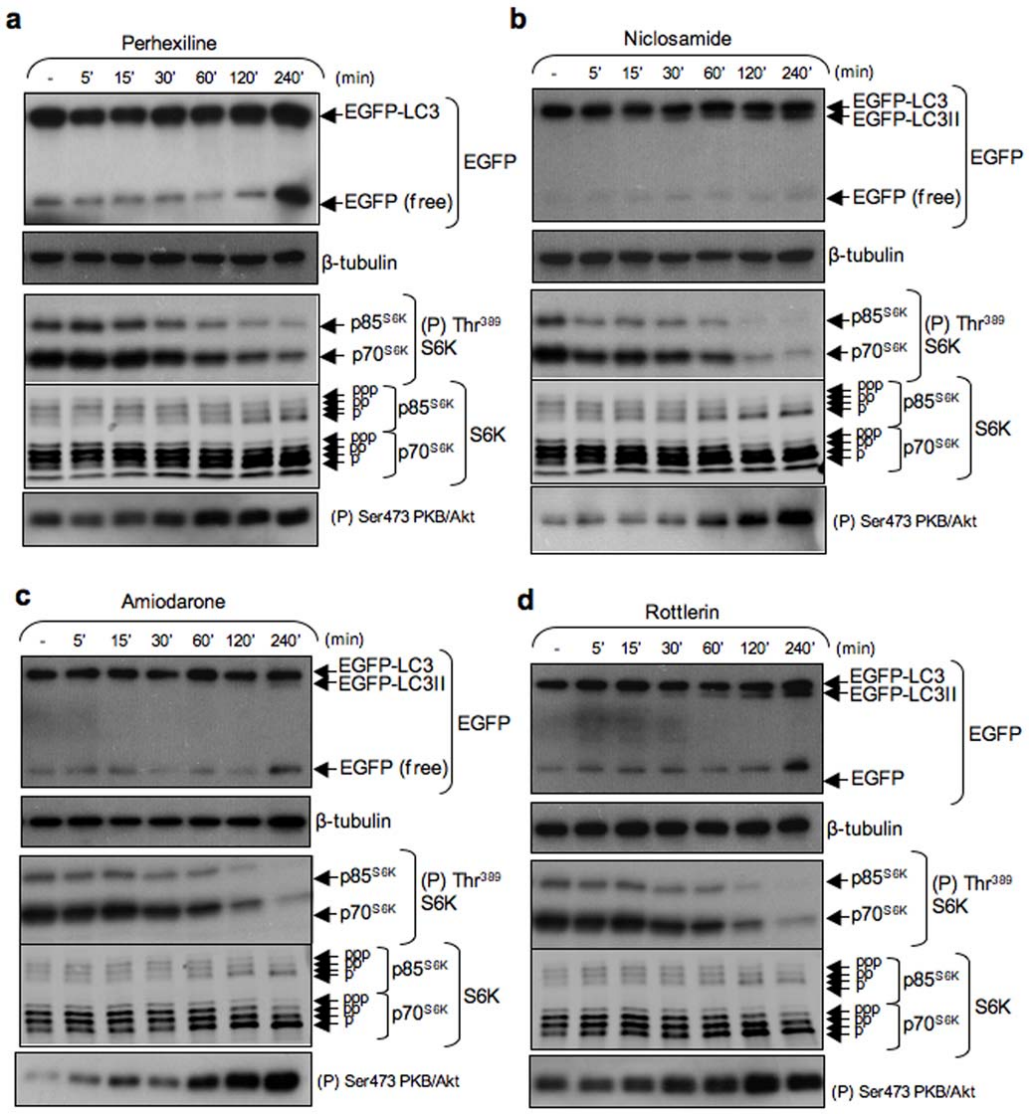
Time-dependent stimulation of EGFP-LC3 processing and inhibition of mTORC1 signaling by perhexiline, niclosamide, amiodarone and rottlerin. MCF-7 cells stably expressing EGFP-LC3 were treated for the indicated times with 10 µM perhexiline, 10 µM niclosamide, 50 µM amiodarone or 3 µM rottlerin. Immunoblotting was carried out using the antisera indicated.

Incubation with perhexiline at varying concentrations (3 to 10 µM) for 4 h caused a concentration-dependent accumulation of free EGFP (Fig. 3, top panel) as well as a small but appreciable increase in EGFP-LC3 lipidation. Niclosamide led to the accumulation of EGFP-LC3II at concentrations as low as 1 µM (Fig. 3, top panel). Free EGFP did not accumulate, but faster migrating bands corresponding to EGFP proteolysis products were readily detectable (Fig. 3). Lipidated EGFP-LC3 (EGFP-LC3II) was detected within 15 min and it continued to accumulate over time (Fig. 4b). Amiodarone also led to the accumulation of EGFP-LC3II (Fig. 4c, 5a) and a substantial increase in free EGFP at 10 µM or higher concentrations (Fig. 3, 5a) within 2 to 4 h (Fig. 4c). Like niclosamide and amiodarone, rottlerin caused the accumulation of EGFP-LC3II and free EGFP, as well as proteolytic fragments of EGFP at 0.3 µM and higher concentrations (Fig. 3). Lipidated EGFP-LC3 was visible within 30 min incubation while free EGFP could be detected between 2 and 4 h (Fig. 4d).

**Figure 5:**
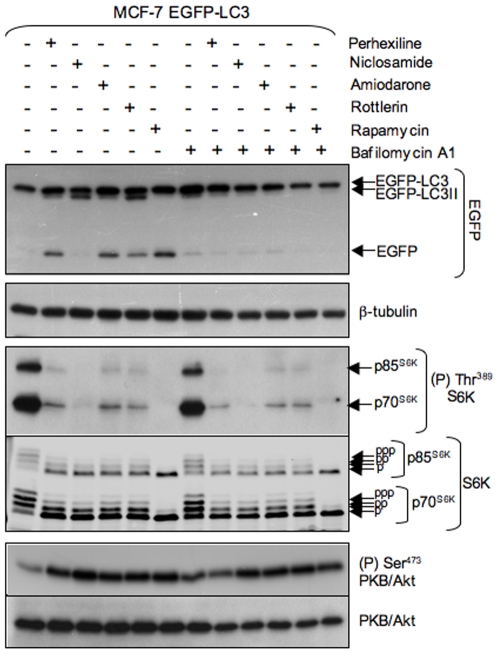
Effect of bafilomycin A1 on EGFP-LC3 processing and inhibition of mTORC1 signaling by perhexiline, niclosamide, amiodarone and rottlerin. MCF-7 cells stably expressing EGFP-LC3 were treated for 4 h with 10 µM perhexiline, 10 µM niclosamide, 50 µM amiodarone or 3 µM rottlerin without or with 100 nM bafilomycin A1.

Bafilomycin A1 is an inhibitor of the vacuolar-type H^+^-ATPase that prevents lysosomal acidification, leading to inhibition of lysosomal hydrolases and fusion of autophagosomes with lysosomes [32]. Cells were next incubated with perhexiline, niclosamide, amiodarone, rottlerin or rapamycin in the absence or presence of bafilomycin A1 and EGFP-LC3 processing and degradation was monitored by western blotting. Bafilomycin A1 clearly blocked the accumulation of free EGFP by the four active chemicals and by rapamycin (Fig. 5), indicating that EGFP-LC3 proteolysis required autophagosome-lysosome fusion and lysosomal hydrolase activity. The observation that all four chemicals stimulated the processing and degradation of EGFP-LC3 in a bafilomycin A1-dependent manner is strong evidence that the chemicals increase autophagic flux and therefore stimulate autophagy.

### Perhexiline, niclosamide, amiodarone and rottlerin inhibit mTORC1 signaling

Autophagy is regulated through both mTORC1-dependent and -independent mechanisms [33]. Since perhexiline, niclosamide, amiodarone and rottlerin stimulate autophagy, we next asked whether any of these chemicals inhibited mTORC1 signaling. mTORC1 phosphorylates S6Ks at Thr^389^ [8]. S6K phosphorylation was completely inhibited by rapamycin, as shown by a disappearance of the phospho-Thr^389^ signal and increased electrophoretic mobility of S6Ks (Fig. S1c). Samples of MCF-7 cells treated with the four chemicals at different concentrations or for different times were analyzed for mTORC1 activation. MCF-7 cells showed robust mTORC1 activation in complete medium containing serum and nutrients (Fig. 3–5). Perhexiline caused strong inhibition of the phosphorylation of p70^S6K^ and p85^S6K^ at 10 µM (Fig. 3) within 30 min and was maximal at 4 h (Fig. 4). Niclosamide caused a strong reduction of S6K phosphorylation at 1 µM and complete inhibition at 3 and 10 µM (Fig. 3). Partial inhibition was detectable as early as 5 min after treatment and was essentially complete after 2 h (Fig. 4). Amiodarone was the least potent of the compounds, with partial inhibition of S6K phosphorylation at 30 µM and complete inhibition at 50 µM (Fig. 3) and this effect was only readily detectable after 2 h (Fig. 4). Rottlerin caused partial inhibition of S6K phosphorylation at 1 µM and complete inhibition at 3 µM (Fig. 3). Inhibition was detectable within 2–4 h (Fig. 4).

**Figure 6:**
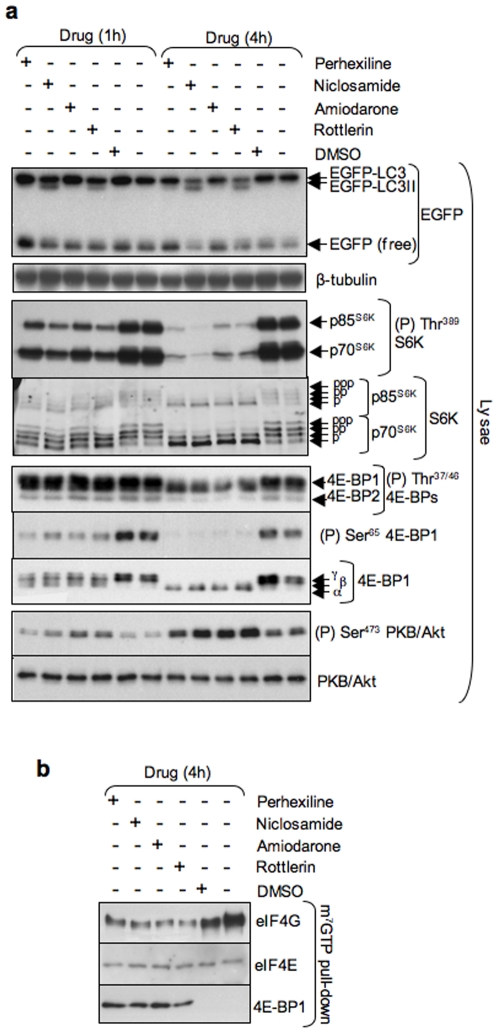
Inhibition of 4E-BP phosphorylation and function by perhexiline, niclosamide, amiodarone and rottlerin. MCF-7 cells stably expressing EGFP-LC3 were incubated with 10 µM perhexiline, 10 µM niclosamide, 50 µM amiodarone, 3 µM rottlerin or DMSO for 1 h or 4 h in complete medium. (a) EGFP-LC3 processing and mTORC1 and mTORC2 activation were monitored by immunoblotting as described in the legend to Fig. 3. Lysates were also probed with antisera to phosphorylated (Thr^37/46^ or Ser^65^) 4E-BPs and total 4E-BP1. (b) eIF4E was purified from cell extracts by m^7^GTP affinity chromatography as described in Materials and Methods and the purified material was probed with antisera against eIF4E, eIF4G and 4E-BP1.

mTORC1 signaling also mediates the phosphorylation of multiple residues on 4E-BP1, including Thr^37/46^ and Ser^65^. Perhexiline, niclosamide, amiodarone and rottlerin, but not DMSO, strongly inhibited phosphorylation at Ser^65^ at 1 h and completely abolished it at 4 h as judged by the decreased binding of phospho-specific antibody and increased electrophoretic mobility of 4E-BP1 (Fig. 6a). These chemicals also decreased the phosphorylation of Thr^37/46^ in 4E-BP1 and 4E-BP2 (Fig. 6a). The phosphorylation of Ser^65^ requires both amino acids and growth factors, whereas phosphorylation of Thr^37/46^ is strongly stimulated by amino acids alone [34]. To examine whether perhexiline, niclosamide, amiodarone and rottlerin inhibited the amino acid-dependent phosphorylation of Thr^37/46^, MCF-7 cells were exposed to perhexiline, rottlerin, amiodarone or niclosamide in medium lacking serum for 1 h or 4 h. All four chemicals reduced the amino acid-mediated phosphorylation of Thr^37/46^ in 4E-BP1 and 4E-BP2, although not completely (Fig. S3b).

Hypophosphorylated 4E-BPs bind to eIF4E thereby precluding the association of eIF4E with eIF4G and the assembly of the eIF4F complex. Phosphorylation of Ser^65^ has been suggested to be particularly important in preventing the re-association of 4E-BP1 with eIF4E [35]. Since the four active chemicals completely block phosphorylation of Ser^65^ in 4E-BP1, we next analyzed their effect on the binding of 4E-BPs to eIF4E by affinity chromatography. MCF-7 cells were propagated in complete medium to near-confluence and then incubated with perhexiline, niclosamide, amiodarone, rottlerin or DMSO (vehicle) for 4 h. Cellular extracts were incubated with 7-methylguanosine-5′-triphosphate beads and the pull-down material probed with antisera against eIF4E, eIF4G and 4E-BP1 in a western blot. In nutrient-rich conditions, where mTORC1 signaling is switched on and 4E-BP1 is hyperphosphorylated, eIF4E associates tightly with eIF4G but not 4E-BP1 (Fig. 6b, lane 6). Inhibition of mTORC1 by rapamycin increases the binding of 4E-BPs to eIF4E with the concomitant release of eIF4G [4]. Similarly, each of the four chemicals increased the binding of 4E-BP1 to eIF4E and partially decreased the association of eIF4G with eIF4E (Fig. 6b).

The four active chemicals and rapamycin also inhibited mTORC1 signaling equally strongly in the absence or in the presence of bafilomycin A1, even though the latter inhibited EGFP-LC3 processing and degradation (Fig. 5). Moreover, bafilomycin A1 did not inhibit mTORC1 signaling (Fig. 5).

Therefore, the four active chemicals inhibit mTORC1 signaling at concentrations that closely parallel those at which they stimulate autophagosome formation as well as EGFP-LC3 processing and degradation. To our knowledge, perhexiline, niclosamide and amiodarone have not previously been shown to inhibit mTORC1 signaling. Rottlerin was previously found to inhibit S6K phosphorylation in rat and cat cardiomyocytes [36], [37]. Perhexiline, amiodarone and rottlerin inhibited mTORC1 signaling much more slowly than rapamycin, which caused complete inhibition within 5 min (not shown), suggesting that they do not inhibit mTORC1 directly. The onset of mTORC1 signaling inhibition by niclosamide was rapid (5 min) but complete inhibition required a longer incubation. The observation that bafilomycin A1 inhibits EGFP-LC3 processing and degradation but that it does not affect the inhibition of mTORC1 signaling by the four active chemicals shows that mTORC1 signaling inhibition is not a consequence of stimulation of autophagy and is consistent with stimulation of autophagy lying downstream of mTORC1 inhibition.

### Perhexiline, niclosamide, amiodarone and rottlerin do not inhibit mTORC2 signaling

mTOR is present in two distinct complexes: mTOR complex 1 (mTORC1) which phosphorylates S6Ks, 4E-BPs and PRAS40 [8], [38]–[40] and mTORC2 which catalyzes the phosphorylation of PKB/Akt and SGK1 [41]–[43]. Insulin receptor substrate-1 (IRS-1), and to a lesser extent IRS-2, protein levels are regulated by S6K1. Hyperactivation of S6K1 signaling leads to transcriptional inhibition of the IRS-1 gene and degradation of IRS-1 and IRS-2 proteins [44]. This is evident in both TSC1 and TSC2 null mouse embryo fibroblasts (MEFs) which exhibit reduced insulin receptor/PI3K signaling and PKB/Akt phosphorylation at Ser^473^ as a result of mTORC1/S6K1 signaling hyperactivation [45]. Prolonged treatment (24 h) of cells that display elevated mTORC1/S6K signaling with rapamycin restores PI3K signaling and PKB/Akt phosphorylation on Ser^473^ [44], [45]. We reasoned that other inhibitors of mTORC1/S6K signaling, such as those identified in this screen, might also increase PKB/Akt phosphorylation. As predicted, MCF-7 cells, which exhibit elevated mTORC1 signaling like TSC1 or TSC2 null MEFs [46], [47], showed increased phosphorylation of Ser^473^ in PKB/Akt when treated with niclosamide, perhexiline, amiodarone or rottlerin (Fig. 3, 6). The increase in PKB/Akt Ser^473^ phosphorylation closely paralleled the decrease in mTORC1 activity as a function of concentration for the four chemicals (Fig. 3). The observation that the four chemicals increased PKB/Akt phosphorylation at Ser^473^ instead of decreasing it shows that they inhibited mTORC1 but not mTORC2 signaling.

### Reversibility of mTORC1 signaling inhibition and autophagosome accumulation

**Figure 7:**
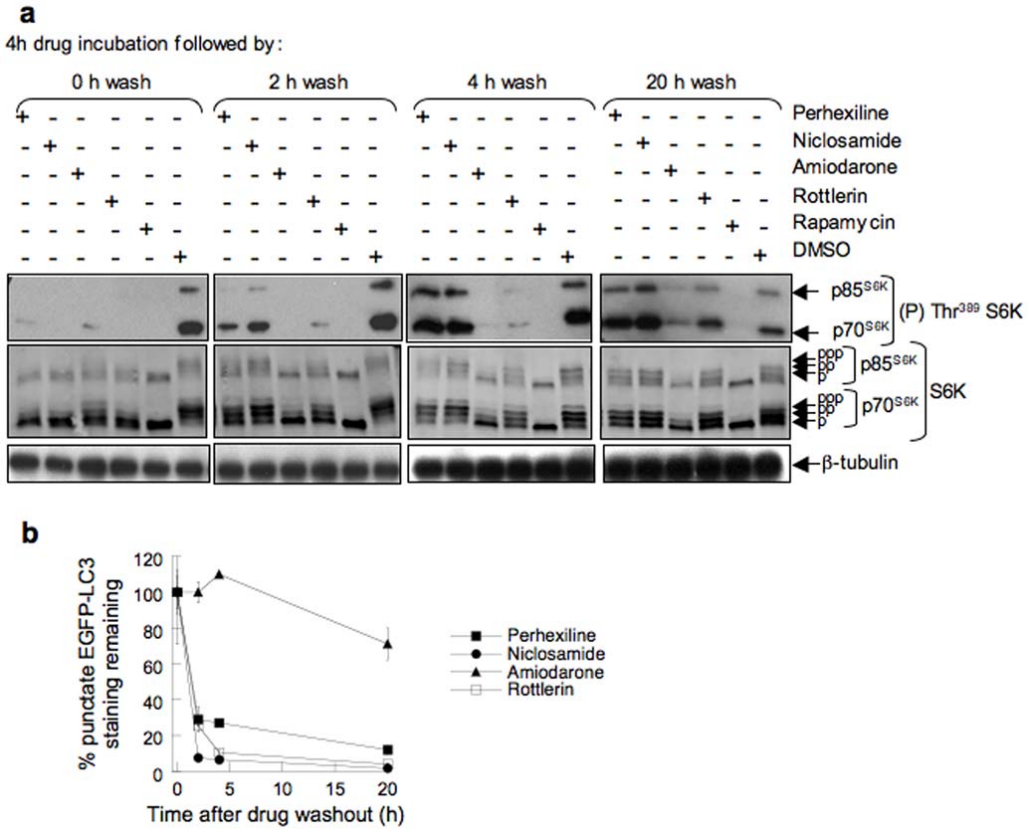
Reversibility of mTORC1 inhibition and EGFP-LC3 punctate fluorescence accumulation. Cells were incubated for 4 h with 10 µM perhexiline, 10 µM niclosamide, 50 µM amiodarone, 3 µM rottlerin, 20 nM rapamycin or DMSO. Drugs were washed away and cells were incubated in drug-free medium for the indicated times. (a) mTORC1 activity was measured by western blotting using phospho-specific and total S6K antibodies. β-tubulin immunostaining was used as a gel loading control. (b) EGFP-LC3 punctate staining was measured using the automated microscopy assay.

MCF-7 cells expressing EGFP-LC3 were incubated with perhexiline, niclosamide, rottlerin, or amiodarone for 4 h in complete medium, the chemicals were washed away and S6K phosphorylation was measured immediately after washing (t = 0) and at 2 h, 4 h and 20 h after. Cells were similarly treated with rapamycin for comparison. All five chemicals inhibited the phosphorylation of p70^S6K^ and p85^S6K^ at Thr^389^ (Fig. 7a), as demonstrated above. Within 2 h following removal of perhexiline or niclosamide, mTORC1 signaling increased substantially and was fully restored by 4 h (Fig. 7a). Inhibition of mTORC1 signaling by rottlerin persisted for 2 h after drug removal but returned to control levels between 4 h and 20 h. By contrast, mTORC1 signaling remained completely inhibited 20 h after removal of amiodarone or rapamycin, indicating that these drugs act essentially irreversibly. Similarly, punctate EGFP-LC3 staining disappeared rapidly upon withdrawal of perhexiline, niclosamide and rottlerin, but not amiodarone, indicating reversible stimulation of autophagy for the former three compounds (Fig. 7b).

### TSC2 is essential only for rottlerin-mediated inhibition of mTORC1 signaling

TSC1/TSC2 plays an important role in the control of mTORC1 signaling. Insulin and growth factors have been proposed to turn on mTORC1 signaling through inactivation of TSC2 [48], [49], which functions as a GTPase-activating protein for the small G-protein Rheb [50]–[53].

**Figure 8:**
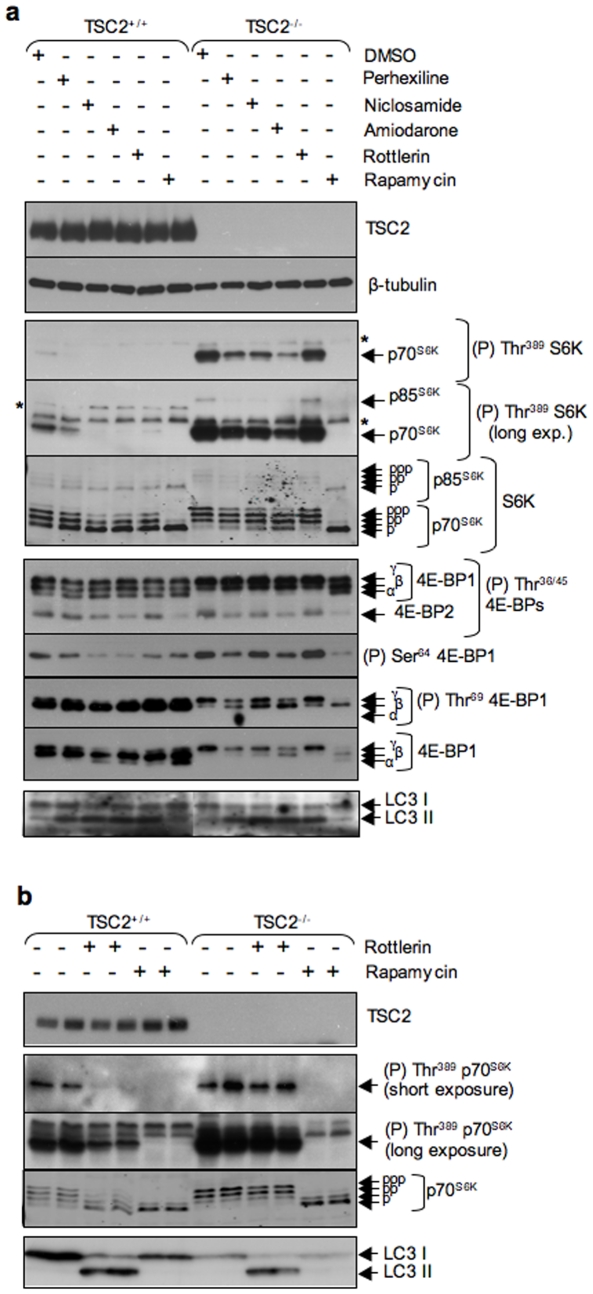
Rottlerin inhibits mTORC1 signaling in a TSC2-dependent manner while perhexiline, niclosamide and amiodarone do not. (a) TSC2^−/−^ or TSC2^+/+^ MEFs were incubated in complete medium with 10 µM perhexiline, 10 µM niclosamide, 3 µM rottlerin, 50 µM amiodarone, DMSO for 4 h or 100 nM rapamycin for 2 h. (b) TSC2^−/−^ or TSC2^+/+^ MEFs were incubated in duplicate in complete medium with 4 µM rottlerin for 1 h, 100 nM rapamycin or DMSO for 30 min. Lysates were analysed by western blotting using the antisera indicated.

Having established that perhexiline, niclosamide, amiodarone and rottlerin inhibit mTORC1 signaling, we sought to determine whether these drugs acted upstream of TSC2. To address this question we made use of TSC2^−/−^ and TSC2^+/+^ mouse embryo fibroblasts also lacking both p53 alleles (hereafter referred to as TSC2^−/−^ and TSC2^+/+^ MEFs) [54]. Near-confluent MEFs were incubated with each of the four chemicals or rapamycin in complete medium for 4 h and lysates were analysed for mTORC1 signaling. As originally reported by Zhang et al. [54], TSC2^−/−^ MEFs exhibited elevated mTORC1 signaling relative to TSC2^+/+^ cells and in both cases rapamycin completely abolished mTORC1 signaling, in accordance with the fact that it inhibits mTORC1 signaling downstream of TSC1/TSC2 (Fig. 8a). Niclosamide, amiodarone and perhexiline inhibited mTORC1 signaling in TSC2^+/+^ as well as in TSC2^−/−^ MEFs (Fig. 8a), indicating that TSC2 is not required for these drugs to inhibit mTORC1 signaling. These results imply that niclosamide, perhexiline and amiodarone inhibit mTORC1 signaling downstream of the tuberous sclerosis complex or through a different signaling cascade.

Rottlerin also inhibited mTORC1 signaling in TSC2^+/+^ MEFs, as judged by the decreased phosphorylation of S6K at Thr^389^ and 4E-BP1 at Ser^64^ (Fig. 8a, b). However, unlike the other drugs, rottlerin failed to inhibit mTORC1 signaling in TSC2^−/−^ cells (Fig. 8a, b), suggesting that it inhibits mTORC1 signaling by activating TSC2. The observation that rottlerin failed to inhibit mTORC1 signaling in TSC2^−/−^ cells provided an opportunity to test whether it induces autophagy through inhibition of mTORC1 signaling. Rottlerin caused LC3II accumulation in both TSC2^+/+^ and TSC2^−/−^ cells, indicating that inhibition of mTORC1 is not required for rottlerin-mediated LC3 processing (Fig. 8a, b).

### Niclosamide, rottlerin and perhexiline selectively kill MEFs in starvation conditions

**Figure 9:**
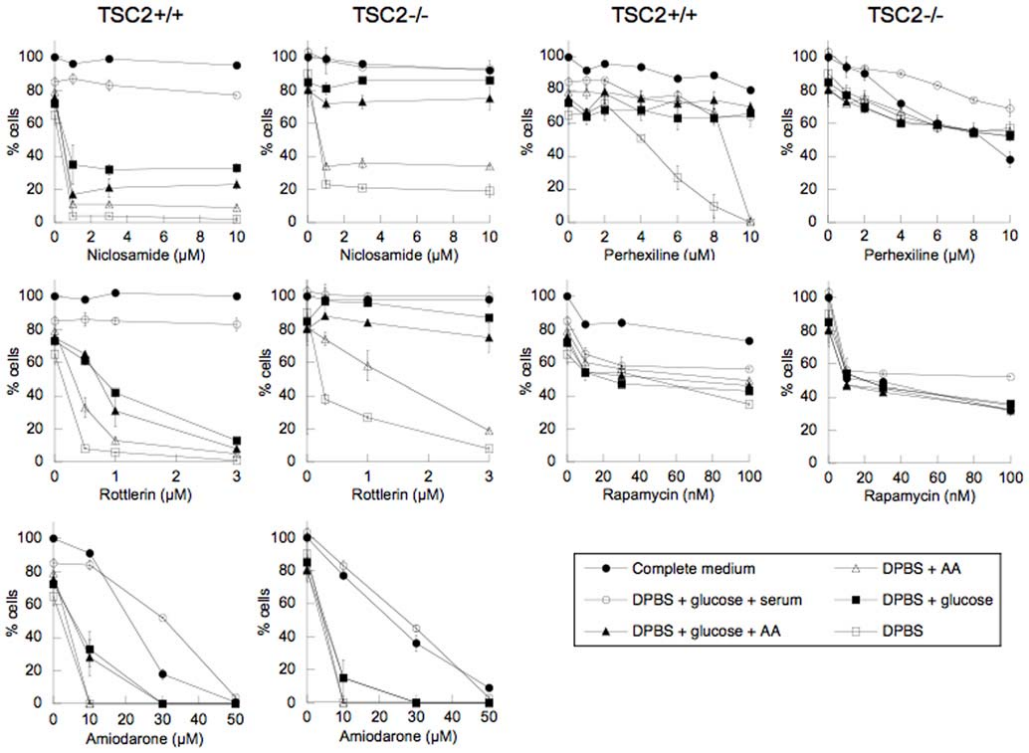
Effect of niclosamide, rottlerin, amiodarone and perhexiline on cell survival in different starvation conditions. TSC2^−/−^ and TSC2^+/+^ MEFs were incubated for 4 h with different concentrations of the chemicals in complete medium or in the different starvation conditions shown. Chemicals and media were washed away and the cells were incubated for 40 h in complete medium without chemicals before measuring cell viability.

Having identified chemicals that stimulate autophagy and inhibit mTORC1 signaling, we next investigated their effect on cell survival and proliferation. TSC2^+/+^ and TSC2^−/−^ MEFs were exposed to varying concentrations of the chemicals in complete medium. After 4 h, the chemicals were removed and the ability of the cells to recover and proliferate was monitored 48 h later. Incubation with niclosamide or rottlerin had no effect on viability or proliferation of MEFs, while perhexiline caused a small reduction in total cell number (Fig. 9). These results indicate that transient inhibition of mTORC1 signaling and stimulation of autophagy in complete medium had no adverse effect on subsequent cell survival or proliferation. Amiodarone was moderately toxic towards MEFs, with an IC_50_ of about 20 µM (Fig. 9), presumably resulting from acting on other cellular targets. Rapamycin decreased the proliferation of TSC2^+/+^ and TSC2^−/−^ cells but did not induce cell death (Fig. 9), consistent with its known cytostatic activity and with its apparently irreversible mTORC1 inhibition [55].

This experiment was repeated using different starvation conditions (Fig. 9). Incubation of MEFs with DPBS (medium lacking glucose, amino acids and serum) for 4 h resulted in a small reduction in cell number at 48 h. Strikingly, addition of niclosamide or rottlerin in DPBS induced complete TSC2^+/+^ cell death. Perhexiline also caused TSC2^+/+^ cell death in DPBS, but only at high concentrations. Amiodarone showed moderate toxicity in complete medium but induced extensive cell death in DPBS. Supplementing DPBS with amino acids did not protect the cells from drug-induced death whereas supplementing with glucose increased survival considerably during exposure to niclosamide, rottlerin or amiodarone, and fully for perhexiline. Addition of both glucose and serum to DPBS rendered cells resistant to niclosamide, rottlerin and perhexiline. By contrast, rapamycin was not any more toxic to cells in DPBS or other starvation conditions than in complete medium.

**Figure 10:**
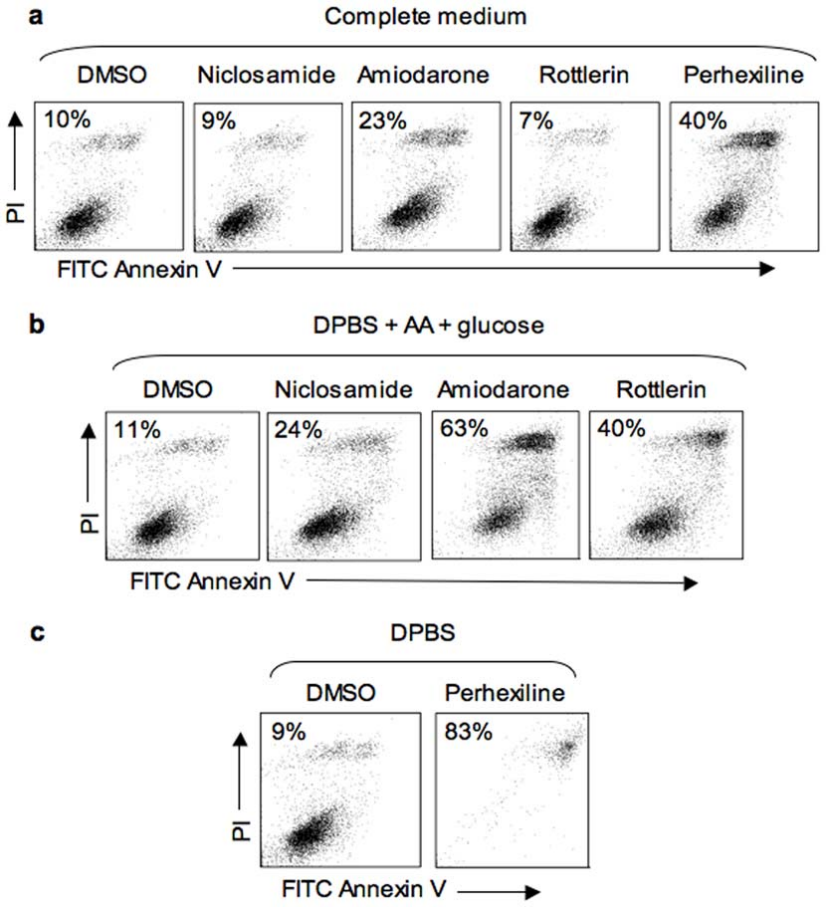
Effect of active chemicals on membrane integrity and apoptosis. TSC2^+/+^ MEFs were incubated for 4 h with DMSO or with 3 µM niclosamide, 30 µM amiodarone, 3 µM rottlerin or 10 µM perhexiline in the cell culture media indicated. Chemicals and media were washed away and the cells were incubated for 24 h in complete medium without chemicals. The extent of cell death and apoptosis was determined by measuring propidium iodide uptake (PI, y axis; relative fluorescence intensity measured in FL2 channel) and FITC Annexin V staining (x axis; relative fluorescence intensity measured in FL1 channel) by flow cytometry. Live cells (PI- and Annexin V-negative) appear in the lower left part of the scatter plots while cells that have died of apoptosis (PI- and Annexin V-positive) appear in the upper right hand part. The numbers in each box indicate the percent of dead or dying (PI- and/or Annexin V-positive) cells.

The observation that some chemicals that inhibit mTORC1 signaling and stimulate autophagy also selectively killed cells in starvation conditions was not anticipated given the established role of autophagy in promoting survival during starvation. Therefore, we next investigated the death mechanism involved. TSC2^+/+^ MEFs were exposed to chemicals for 4 h in complete medium or in starvation conditions. The drugs were then washed away and after 24 h, the cells were stained with FITC-Annexin V and propidium iodide. Annexin V binds to phosphatidylserine on the outer leaflet of the plasma membrane and labels cells actively undergoing apoptosis while propidium iodide is excluded by viable cells with an intact plasma membrane and therefore labels dead cells. Flow cytometry analysis revealed that in starvation conditions, the four chemicals caused a considerable increase in cells labeled with FITC-Annexin and propidium iodide (Fig. 10). Autophagic cell death is not associated with increased Annexin V binding [56], indicating that the chemicals trigger apoptotic cell death in starvation conditions.

### TSC2 is an important determinant of cellular sensitivity to active chemicals in starvation conditions

TSC2^+/+^ and TSC2^−/−^ MEFs responded similarly to each of the four chemicals in complete medium but not in starvation conditions (Fig. 9). TSC2^−/−^ cells were generally more resistant than TSC2^+/+^ MEFs to exposure to niclosamide, rottlerin and perhexiline in the different starvation conditions. For example, exposure of TSC2^−/−^ cells to niclosamide or rottlerin in DPBS strongly decreased cell survival but addition of glucose was sufficient to almost completely rescue TSC2^−/−^ cell survival, whereas both glucose and serum were necessary to prevent killing of TSC2^+/+^ by these chemicals (Fig. 9). By contrast, the effect of rapamycin was similar in TSC2^+/+^ and TSC2^−/−^ cells under all conditions, consistent with it acting downstream of TSC1/TSC2. These results show that the tuberous sclerosis complex plays an important role in the selective killing of cells by niclosamide, rottlerin and perhexiline in starvation conditions.

## Discussion

This study identifies four chemicals (perhexiline, niclosamide, amiodarone and rottlerin) that stimulate autophagy and inhibit mTORC1 signaling within a few hours in conditions of nutrient and growth factor sufficiency, under which autophagy is normally downregulated and mTORC1 signaling switched on. Each of the four chemicals showed interesting similarities to and differences from the well-characterized mTORC1 inhibitor rapamycin. Rapamycin inactivates mTORC1 very rapidly, within a few minutes of cellular exposure. Niclosamide also rapidly inhibits mTORC1 signaling but this inhibition is initially partial, complete inhibition being achieved after 1–2 h incubation. The three other chemicals required 1–2 h incubation to inhibit mTORC1 signaling, strongly implying that they do not inhibit mTORC1 directly, but target upstream mTORC1 control pathways. Rapamycin is highly potent, complete mTORC1 inhibition being achieved at low nanomolar concentrations. Niclosamide is also potent, with sub-micromolar activity while the other three chemicals inhibit mTORC1 at micromolar concentrations. Rapamycin inhibits mTORC1 independently of TSC1/TSC2, similar to amiodarone, perhexiline and niclosamide. By contrast, rottlerin can only inhibit mTORC1 signaling in TSC2^+/+^ cells, implying that it inhibits mTORC1 signaling upstream of TSC2. All four compounds selectively inhibit mTORC1 but not mTORC2 signaling, as does rapamycin.

Notably, the chemicals identified in this study differ from rapamycin with respect to the reversibility of mTORC1 inhibition. Rapamycin inhibits mTORC1 signaling irreversibly [55]. By contrast, inhibition of mTORC1 signaling by niclosamide, perhexiline and rottlerin is reversed upon drug removal, while amiodarone is only slowly reversible. Pharmacologically, reversible inhibition is considered a favorable property, especially for drug targets whose activity is necessary for normal cellular functions, because prolonged inhibition caused by irreversible inhibitors can lead to severe side effects [57]. This property should facilitate the fine-tuning of chemical inhibition of mTORC1 signaling in cells or animals for studies of mechanism of action or therapeutic potential.

The effects of transient exposure on cell proliferation and viability between the four compounds and rapamycin also differed considerably. Transient exposure to nanomolar concentrations of rapamycin caused long-lasting inhibition of cell proliferation, consistent with its irreversible mode of mTORC1 inhibition. By contrast, 4 h incubation with niclosamide, rottlerin and perhexiline at concentrations that were sufficient to profoundly inhibit mTORC1 signaling and stimulate autophagy had little or no effect on cell viability or proliferation in cell culture medium containing nutrients and serum. This result is consistent with the reversible nature of mTORC1 signaling inhibition by these chemicals and demonstrates that strong but transient inhibition of mTORC1 signaling and stimulation of autophagy are not deleterious to cells. The observation that amiodarone killed cells while niclosamide, perhexiline, rottlerin and rapamycin did not suggests that amiodarone acts on targets other than mTORC1 and autophagy to induce toxicity.

The effects of short exposure to the four chemicals on cell survival and proliferation in starvation conditions also differed from those of rapamycin. Transient exposure to rapamycin did not kill cells but was cytostatic and affected equally cells in complete medium and in starvation conditions. By contrast, the four autophagy-stimulating chemicals all enhanced to varying degrees cell killing in starvation conditions, with niclosamide and rottlerin showing the most pronounced effect. Killing was rescued partially by glucose and totally by further addition of serum, indicating that an interplay between energy status sensing, growth factor signaling and drug action is important for cell death. This observation was unexpected because autophagy is a well-established survival response to starvation and we anticipated that stimulators of autophagy would increase cell survival in starvation conditions. However, a form of death termed type II or autophagic death has been attributed to unregulated autophagy [56], [58], [59]. It can be suggested that simultaneous exposure to multiple autophagy stimuli might overactivate autophagy and transform a normally protective response into a death mechanism. However this does not appear to be the case because dying cells showed the presence of phosphatidylserine on the outer leaflet of their plasma membrane, indicating that death occurred through apoptosis. The observation that TSC2^−/−^ cells are very significantly, but not completely, protected from death in starvation firmly implicates the TSC1/TSC2 signaling cascade in the death mechanism. The interesting observation that rapamycin does not trigger cell death in starvation but that upstream inhibitors of mTORC1 signaling do indicates that death does not result from mTORC1 inhibition per se. Rather, it implies the involvement of a TSC2-dependent but mTORC1-independent cell survival pathway. Lee et al [60] showed that loss of TSC2 activates p53 and increases cell death in response to glucose starvation and that inactivation of mTORC1 by rapamycin protects TSC2 mutant cells from starvation-induced death. They did not observe this effect in TSC2^−/−^ p53^−/−^ cells, which responded similarly to rapamycin treatment and were as resistant to glucose starvation as TSC2^+/+^ p53^−/−^ cells. Since TSC2^−/−^ p53^−/−^ cells were used in the present study, the death pathway we speculate upon must be distinct from that identified by Lee at al. [60].

Perhexiline, niclosamide, amiodarone and rottlerin most likely inhibit mTORC1 signaling by acting on upstream regulatory pathways, unlike the recently described inhibitors of mTORC1/2 Torin1 [61] and Ku-0063794 [62] and the dual PI3k/mTOR inhibitors PI-103 [63] and NVP-BEZ235 [64], which inhibit these kinases directly.

Rottlerin is a widely used pharmacological agent believed until recently to inhibit PKC∂ selectively. However, it has now been unequivocally shown that rottlerin does not inhibit this kinase. Rather, it inhibits potently several other kinases and enzymes including malate dehydrogenase, activates several types of K^+^ channels, and uncouples mitochondrial oxidative phosphorylation [65]. Consistent with its uncoupling activity, rottlerin has been reported to reduce cellular ATP levels, causing AMPK activation through a poorly understood signaling mechanism involving the tumor suppressor LKB1 [66]. AMPK phosphorylates and activates TSC2 to switch off mTORC1 signaling [67]. It is tempting to speculate that rottlerin inhibits mTORC1 signaling through the phosphorylation of Ser 1345 on TSC2 by AMPK. However, there are currently no antibodies available to study this phosphorylation on TSC2. Although it is possible that rottlerin stimulates autophagy via AMPK, TSC2 and mTORC1, this is unlikely to be the only mechanism because LC3 processing still occurs in TSC2^−/−^ cells in which rottlerin does not inhibit mTORC1 signaling.

Niclosamide is a salicylanilide antihelmintic drug that was approved for use in humans nearly 50 years ago [68], [69]. It was developed on the basis of activity in rodent models of parasitic worm infection rather than inhibition of a precise cellular target and its mode of action remains unclear. Niclosamide is believed to owe its antiparasitic effects to protonophoric activity [69], the ability of some chemicals to embed themselves within membranes and, via a continuous cycle, carry protons across membranes along their concentration gradient. Niclosamide and analogues inhibit glucose uptake by parasites, possibly by lowering the plasma membrane potential of tegument cells through protonophoric activity [70], [71]. Niclosamide can also uncouple mitochondrial oxidative phosphorylation in worms [72] but this is not considered relevant to antihelmintic activity in the anaerobic intestinal environment [73]. Niclosamide can also uncouple mitochondrial oxidative phosphorylation in human cells [74], [75], raising the possibility that it inhibits mTORC1 signaling and stimulates autophagy by lowering ATP levels in the cell. However, this is not the case because niclosamide treatment did not significantly decrease cellular ATP concentration (96.6±0.3% in niclosamide-treated cells versus 100.0±3.3% in control cells treated with DMSO) during 4 h incubation, and mTORC1 inhibition by niclosamide did not require TSC2.

Amiodarone is an antianginal and antiarrhythmic drug [76] that exerts many pharmacological activities including blockage of multiple ion channels [77]. Interestingly, exposure of yeast to amiodarone in nutrient-rich medium causes a rapid change in gene expression pattern resembling that elicited by starvation and by rapamycin, prompting the authors to suggest that amiodarone interferes with nutrient sensing and regulatory networks by an uncharacterized mechanism [78]. Amiodarone inhibited mTORC1 in a TSC2-independent manner and killed cells in starvation conditions in a manner that was not affected by TSC2, suggesting that its mechanism of action differs from that of rottlerin or niclosamide.

Perhexiline is an antianginal drug with multiple pharmacological activities [79]. It was originally designated as a calcium channel blocker but it shows no such activity at therapeutic concentrations [80], [81]. Rather, there is increasing evidence that it acts by inhibiting carnitine palmitoyltransferase, an enzyme that permits the entry of fatty acids into mitochondria [80]. This inhibition shifts myocardial substrate utilization from fatty acids to lactate and glucose, which increases ATP generation per unit oxygen consumed and exerts an oxygen sparing effect on the heart muscle[80], [82], [83]. No protonophoric, mitochondrial uncoupling, or protein kinase inhibition activity has been attributed to this drug. Perhexiline inhibited mTORC1 in a TSC2-independent manner but its effects in starvation were not as pronounced as those of rottlerin, niclosamide or amiodarone.

The four chemicals identified in this study should be useful pharmacological tools to manipulate mTORC1 signaling and autophagy in cells and in animal models of disease. Perhexiline can be administered continuously to humans for many years, with mean plasma concentrations of ∼2 µM without any significant adverse effects [80], [84]. Severe side effects do not occur at serum concentrations below 5 µM [84]. Perhexiline induced autophagosome accumulation in the 1–3 µM range and strong mTORC1 inhibition was seen at 10 µM during 4 h exposure, close to therapeutic concentrations. Niclosamide exerts its antiparasitic activity in the intestinal lumen and was not designed to be absorbed through the intestine. Nevertheless, it shows 10% oral bioavailability and micromolar serum concentrations are achieved after a single oral dose in animals or humans [85], [86]. Intravenous administration of niclosamide to rats gave rise to a peak plasma concentration of 25 µM [86]. Niclosamide very strongly inhibited mTORC1 signaling at concentrations as low as 1 µM. Therefore, therapeutic inhibition of mTORC1 signaling may be achievable using niclosamide or a derivative. Amiodarone can be administered safely for many years with a mean steady state plasma concentration of 2 µM. Peak plasma concentrations can be as high as 60 µM [87]. Amiodarone inhibited mTORC1 signaling at concentrations above 10 µM. Rottlerin is not an approved drug but it shows a low toxicity profile in rodents [88] and it inhibits mTORC1 signaling at 1 µM.

The observation that drugs already approved for human use can reversibly inhibit mTORC1 and stimulate autophagy *in vitro* at concentrations that correspond to or are close to those observed in the circulation during treatment should greatly facilitate the preclinical and clinical testing of mTORC1 inhibition in indications such as tuberous sclerosis, diabetes, cardiovascular disease, protein misfolding diseases and cancer.

## Materials and Methods

### Chemicals

Cell culture reagents were purchased from Invitrogen, unless stated otherwise. General laboratory chemicals were purchased from Sigma-Aldrich, Fisher Scientific and BDH Inc. The 3,584 chemicals used in the screen were composed of chemicals from the Prestwick, Sigma LOPAC, Microsource Spectrum and Biomol natural products collections, and provided by the Canadian Chemical Biology Network (www.ccbn-rcbc.ca). Perhexiline, amiodarone, niclosamide, rottlerin, chloroquine and bafilomycin A1 were also purchased from Sigma-Aldrich. Rapamycin was from Calbiochem, Hoechst 33342 from Invitrogen and paraformaldehyde from BDH.

### Plasmids and antibodies

The pEGFP-LC3 vector, previously described by Kabeya et al. [22], was a kind gift of Dr. Tamotsu Yoshimori (Osaka, Japan). pRK7 FLAG-tagged human TSC2 [48], was a generous gift from Dr. Andrew Tee (Cardiff, UK). Anti-4E-BP1 antibody was prepared by Dr. Josep Parra and is described in Wang et al. [34]. Anti-phospho Thr^37/46^ 4E-BP1 (#9459), anti-phospho Thr^70^ 4E-BP1 (#9455), anti-phospho Thr^389^ S6K (#9205), anti-phospho Ser^473^ Akt (#9271), anti-Akt (#9272), anti-eIF4E (#9742) and anti-eIF4G (#2498) antibodies were bought from Cell Signaling Technology (Massachusetts, USA). Anti-S6K C-18 (#230), anti-MAP LC3 H-50 (#28266), anti-TSC2 C-20 (#893), anti-β-tubulin H-235 (#9104) and anti-phospho Ser^65^ 4E-BP1 (#18091) antibodies were purchased from Santa Cruz Biotechnology, Inc. Anti-GFP antibody (#1814460) was from Roche. Anti-LC3 antibody was from Nanotools (#0260-100/LC3-2G6).

### Cell culture, transfection and starvation procedures

TSC2^−/−^/p53^−/−^ and TSC2^+/+^/p53^−/−^ MEFs were a generous gift of Dr. David Kwiatkowski (Massachusetts, USA) and have been previously described by Zhang et al. [54]. These cells were maintained in high glucose Dulbecco's modified Eagle's medium (DMEM) supplemented with 10% (v/v) fetal bovine serum, 100 units/ml penicillin/streptomycin and 2 mM L-glutamine (Sigma-Aldrich). MCF-7 (Michigan Center Foundation line 7) cells were transfected with pEGFP-LC3 using Effectene transfection reagent (#301425, Invitrogen) according to the manufacturer's specification. Stably-transfected cells were selected for 2 months in RPMI-1640 medium supplemented with 400 µg/ml G418 (#V7982, Promega), 100 units/ml penicillin/streptomycin, 1 mM HEPES, 10% (v/v) foetal bovine serum and 2 g/L sodium bicarbonate. EGFP-LC3 expressing cells were sorted by fluorescence-activated cell sorter and pooled. The pooled cells were thereafter maintained in the RPMI-1640 medium described above and used for all experiments. For starvation experiments, cells were rinsed twice in DPBS (#14040, Invitrogen) before incubations in the different starvation media. Serum starvation was carried out by incubating cells in their corresponding medium lacking fetal bovine serum. Glucose starvation was carried out by incubating cells in Hank's Balanced Salt Solution (#37150, StemCell Technologies) or DPBS supplemented with amino acids from a 10x mixture [39] and 10% (v/v) dialysed foetal bovine serum. For amino acid starvation, cells were incubated for 4 h in either DPBS containing 11 mM glucose. Starvation of serum, glucose and amino acids was carried out by incubating cells in DPBS.

### Chemical screen for modulators of autophagy

MCF-7 cells stably expressing EGFP-LC3 were seeded in PerkinElmer View 96-well plates at 20,000 cells per well. Eighteen hours after seeding, chemicals were added to each well to a final concentration of ∼15 µM using a Biorobotics Biogrid II robot equipped with a 0.7 mm diameter 96-pin tool. Plates were incubated for 4 h at 37°C. The medium was then removed and the cells were fixed with 3% (v/v) paraformaldehyde containing 500 ng/ml Hoechst 33342 for 15 min at room temperature. Fixed cells were washed once with PBS containing 1 mM MgCl_2_ and 0.1 mM CaCl_2_ and then stored in the same medium at 4°C until plates were ready for analysis. Plates were read in a Cellomics™Arrayscan V^TI^ automated fluorescence imager. Cells were photographed using a 20x objective in the Hoechst and GFP (XF-100 filter) channels. The compartment analysis algorithm was used to identify the nuclei, apply a cytoplasmic mask and quantitate GFP spots in the GFP channel fixed at 250 pixel intensity units. Fluorescence intensity in the GFP channel was gated at 10 average pixel intensity units inside the cytoplasmic mask to select against cells expressing very low levels of EGFP-LC3. The total pixel intensity for punctate EGFP-LC3 was acquired as ‘circ spot total intensity ch2’. Wells showing 3-fold or higher increase in punctate EGFP-LC3 staining over control were re-examined visually to eliminate any false positives resulting from precipitation of fluorescent compounds. Compounds inducing a 3-fold or higher increase in punctate EGFP-LC3 staining were considered ‘hits’. Compounds causing a decrease in cell number to fewer than 4,000/well were disregarded as toxic chemicals. The Z-factor of the assay [26] was determined from punctate EGFP-LC3 measured in cells treated with DMSO (negative control) or chloroquine (positive control).

### Cell lysis and protein quantitation

Cells were harvested in the following extraction buffer: 20 mM Tris-HCl pH 7.5, 150 mM NaCl, 1 mM EDTA, 1 mM EGTA, 1% (v/v) Triton X100, 2.5 mM sodium pyrophosphate, 1 mM β-glycerophosphate supplemented with fresh 1 mM Na_3_VO_4_, 1 mM dithiothreitol and 1x complete protease inhibitor cocktail (#11697498001, Roche Molecular Biochemicals). Lysates were pre-cleared by centrifugation at 18,000 g for 15 min at 4°C. Supernatant was collected and protein quantitated using the BCA protein assay kit (#23227, Pierce).

### Viability/proliferation assay

Cells were seeded in 96-well plates at 8000 cells per well and grown for 18 h. Cells were then treated with chemicals in different starvation media for 4 h as described above. The drugs and media were then removed and cells were incubated in complete medium without drugs. Cell viability/proliferation was measured 48 h later using the (3-[4,5-Dimethylthiazol-2-yl]-2,5-diphenyl-tetrazolium bromide (MTT) assay (M2128, Sigma) as described [89].

### Flow cytometry

After drug treatment, the cell culture medium was collected to retain floating cells and attached cells were dislodged using EDTA. Floating and attached cells were combined and harvested by centrifugation. The cell pellets were suspended in 100 µl binding buffer (10 mM HEPES pH 7.4, 140 mM NaCl, 2.5 mM CaCl_2_) and incubated with 5 µl FITC Annexin V (BD Biosciences) and 10 µl of a propidium iodide solution (50 µg/ml; Sigma) for 15 min in the cold. Staining for Annexin V and propidium iodide was assessed by flow cytometry on a FACSCalibur instrument (BD Biosciences) followed by data analysis using FlowJo software (Tree Star Inc).

### SDS-PAGE and western blot

EGFP-LC3 processing was assayed by western blot as described [90]. TSC2 protein levels and S6K phosphorylation were analyzed by separating protein samples on a 10% acrylamide gel containing 0.1% methylene bisacrylamide. Endogenous LC3 lipidation and 4E-BP1 phosphorylation were assessed by resolving protein samples on a 13.5% acrylamide gel with 0.36% methylene bisacrylamide. Resolved protein was subjected to electroblotting onto polyvinylidene fluoride membrane, blocked with 5% (w/v) fat-free milk and incubated with antisera as indicated. Endogenous LC3 and 4E-BP1 proteins were cross-linked to the membrane with 0.2% (v/v) glutaraldehyde in PBS-0.02% (v/v) Tween20 for 30 min, following transfer and prior to the blocking step.

### m^7^GTP affinity chromatography

200 µg of protein lysate from MCF-7 cells stably expressing EGFP-LC3 were incubated with 30 µl (neat) 7-methylguanosine-5′-triphosphate sepharose 4B (m^7^GTP) (#27-5025-01, GE Healthcare) for 90 min at 4°C, mixing end-over-end. Beads were then pelleted by centrifugation at low speed for 30 sec. Beads were washed twice with extraction buffer supplemented with protease and phosphatase inhibitors and dithiothreitol as described in the *Cell lysis and protein quantitation* section. Material bound to the m^7^GTP sepharose was then eluted by boiling for 10 min with 50 µl 5x sample buffer (200 mM Tris-HCl pH 6.8, 8% (w/v) sodium dodecyl sulphate, 0.4% (w/v) bromophenol blue, 40% (v/v) glycerol) containing 100 mM dithiothreitol. Eluted material was analysed by SDS-PAGE/immunoblot with anti-4E-BP1, anti-eIF4G and anti-eIF4G antibodies.

## Supporting Information

Figure S1 Stimulation of punctate EGFP-LC3 accumulation and EGFP-LC3 processing by rapamycin. (a) MCF-7 cells expressing EGFP-LC3 were incubated with 20 nM rapamycin for the indicated times and images were acquired using the automated microscopy assay. (b) MCF-7 cells expressing EGFP-LC3 were treated in complete medium with 20 nM rapamycin for the indicated times. EGFP-LC3 processing was monitored by probing lysates with anti-GFP antibody and mTORC1 activity by probing lysates with antisera against phosphorylated (Thr389) S6K and total S6K. (c) Quantitation of punctate EGFP-LC3 staining during 4 h incubation in complete medium or amino acid and serum starvation using the screening assay.
(0.71 MB TIF)

Figure S2 Illustration of screening assay and results. (a) Data used for determination of Z-factor. Forty-eight wells were treated for 4 h with DMSO (negative control) and 48 wells were treated with chloroquine (positive control). Punctate EGFP-LC3 staining was determined using the screening assay. (b) Results from one 96-well plate of screening chemicals demonstrating quantitation of punctate EGFP-LC3 staining, with four positive controls (chloroquine) and two active chemicals indicated by blue arrows. The right panels show images obtained from the automated microscopy screen for an inactive chemical (top) and an active chemical (bottom).
(0.25 MB TIF)

Figure S3 Niclosamide, rottlerin, amiodarone and perhexiline inhibit the amino acid-dependent phosphorylation of 4E-BP1 at Thr37/46. MCF-7 cells stably expressing EGFP-LC3 were incubated in Hank's balanced salt solution supplemented with 10% (v/v) dialysed serum for 1 h or 4 h. Where indicated, cells were simultaneously incubated with 10 µM perhexiline, 10 µM niclosamide, 50 µM amiodarone, 3 µM rottlerin or 0.2% (v/v) DMSO for the times indicated. (a) Lysates were probed for EGFP-LC3 processing using GFP antibody. Tubulin staining was used as a loading control. (b) Lysates were probed for 4E-BP phosphorylation at Thr37/46 or total 4E-BP1 levels using the antisera indicated.
(0.16 MB TIF)
