# Supplementary figures and images for: Screen for Chemical Modulators of Autophagy Reveals Novel Therapeutic Inhibitors of mTORC1 Signaling
Source: PLoS One. 2009 Sep 22;4(9):e7124. doi: 10.1371/journal.pone.0007124 (PMC2742736; doi:10.1371/journal.pone.0007124)

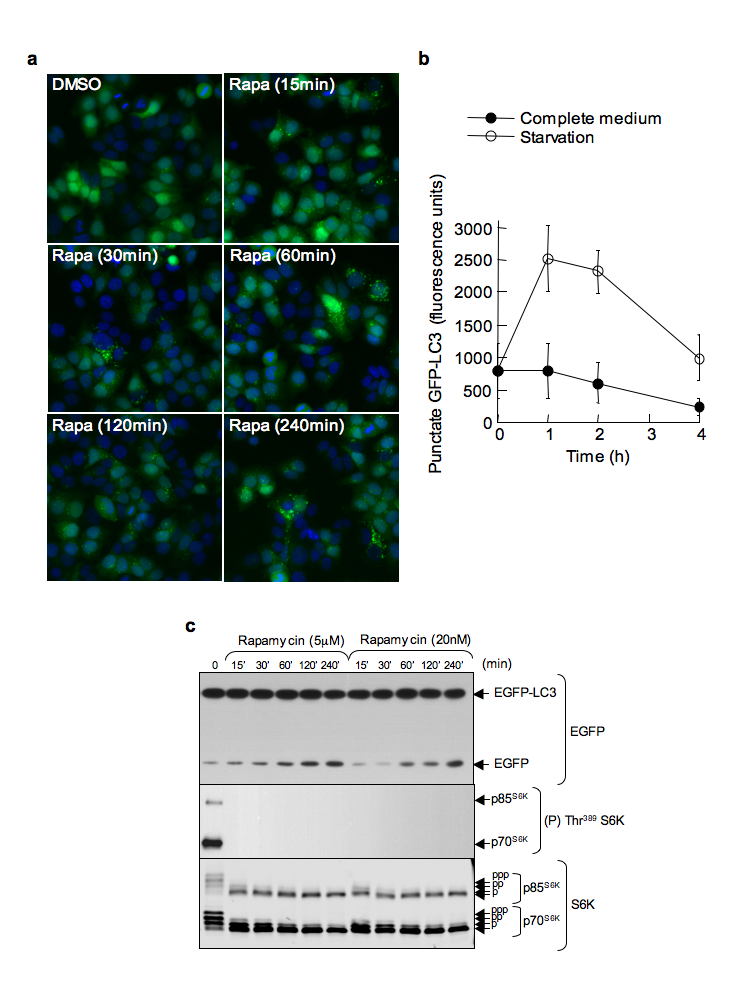

Supplement: Figure S1 — Stimulation of punctate EGFP-LC3 accumulation and EGFP-LC3 processing by rapamycin. (a) MCF-7 cells expressing EGFP-LC3 were incubated with 20 nM rapamycin for the indicated times and images were acquired using the automated microscopy assay. (b) MCF-7 cells expressing EGFP-LC3 were treated in complete medium with 20 nM rapamycin for the indicated times. EGFP-LC3 processing was monitored by probing lysates with anti-GFP antibody and mTORC1 activity by probing lysates with antisera against phosphorylated (Thr389) S6K and total S6K. (c) Quantitation of punctate EGFP-LC3 staining during 4 h incubation in complete medium or amino acid and serum starvation using the screening assay. (0.71 MB TIF) [file pone.0007124.s001.tif]

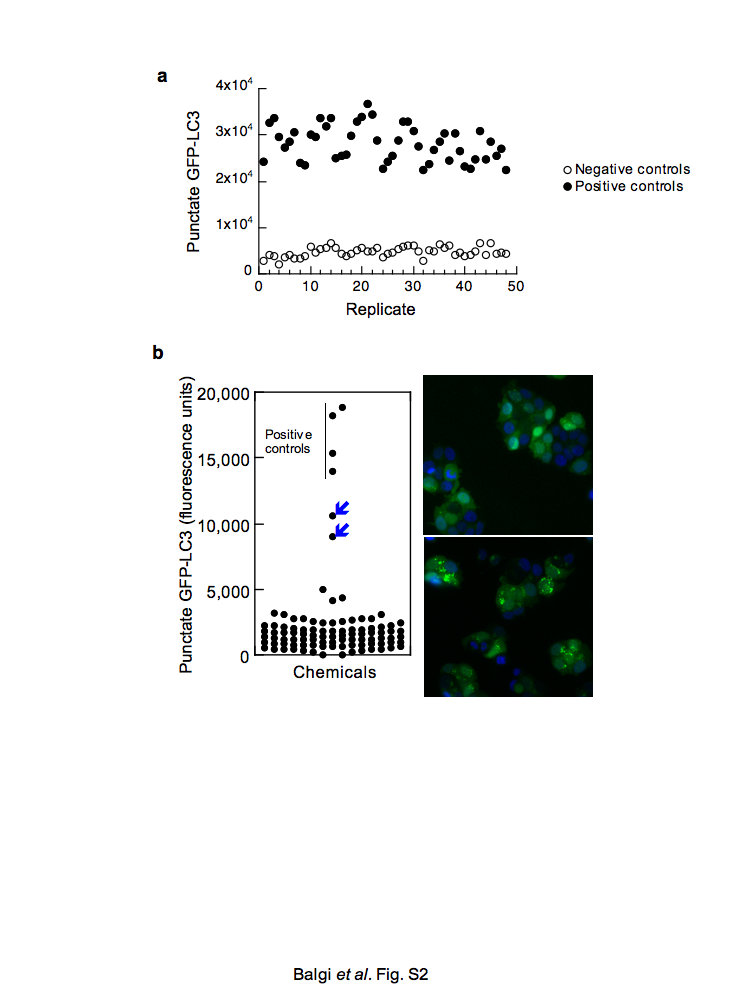

Supplement: Figure S2 — Illustration of screening assay and results. (a) Data used for determination of Z-factor. Forty-eight wells were treated for 4 h with DMSO (negative control) and 48 wells were treated with chloroquine (positive control). Punctate EGFP-LC3 staining was determined using the screening assay. (b) Results from one 96-well plate of screening chemicals demonstrating quantitation of punctate EGFP-LC3 staining, with four positive controls (chloroquine) and two active chemicals indicated by blue arrows. The right panels show images obtained from the automated microscopy screen for an inactive chemical (top) and an active chemical (bottom). (0.25 MB TIF) [file pone.0007124.s002.tif]

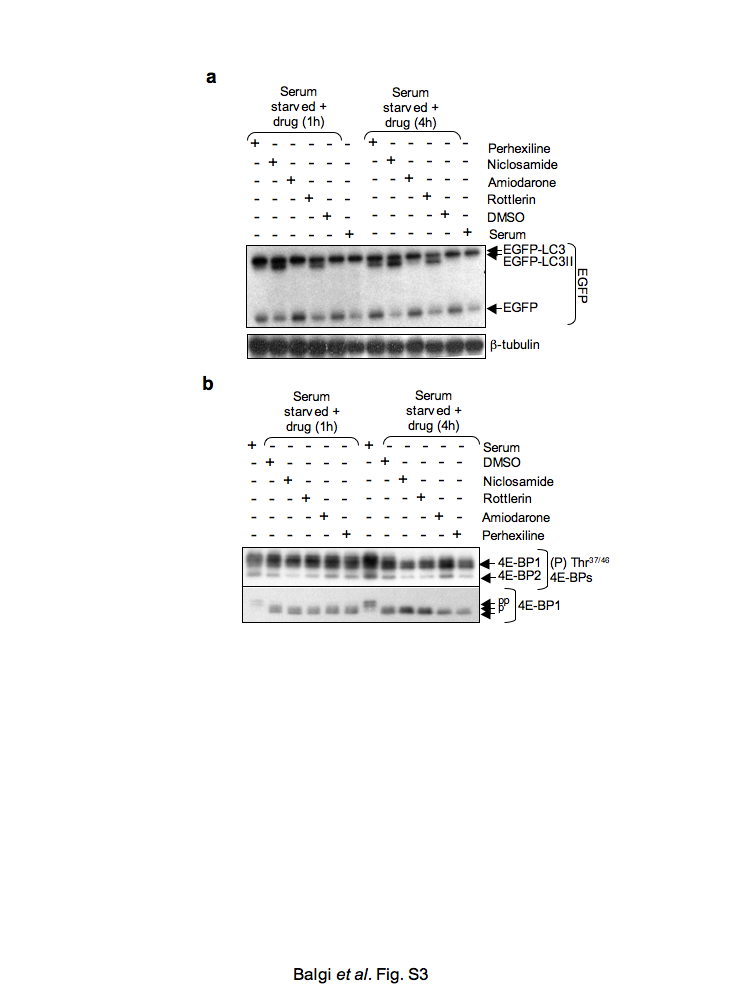

Supplement: Figure S3 — Niclosamide, rottlerin, amiodarone and perhexiline inhibit the amino acid-dependent phosphorylation of 4E-BP1 at Thr37/46. MCF-7 cells stably expressing EGFP-LC3 were incubated in Hank's balanced salt solution supplemented with 10% (v/v) dialysed serum for 1 h or 4 h. Where indicated, cells were simultaneously incubated with 10 µM perhexiline, 10 µM niclosamide, 50 µM amiodarone, 3 µM rottlerin or 0.2% (v/v) DMSO for the times indicated. (a) Lysates were probed for EGFP-LC3 processing using GFP antibody. Tubulin staining was used as a loading control. (b) Lysates were probed for 4E-BP phosphorylation at Thr37/46 or total 4E-BP1 levels using the antisera indicated. (0.16 MB TIF) [file pone.0007124.s003.tif]
